# Diagnostic accuracy of UACR in CKD screening among diabetics: A community study

**DOI:** 10.6026/973206300222863

**Published:** 2026-05-31

**Authors:** Utsav Haldar, Niranjan Gopal, Anand Chellappan, Dnyanesh Amle, Nandeesh Kuravatti, Neha Patel, Jyoti E John, Deepa Telgote

**Affiliations:** 1 Department of Biochemistry, All India Institute of Medical Sciences, Nagpur, Maharashtra, India;; 2 Department of Nephrology, All India Institute of Medical Sciences, Nagpur, Maharashtra, India;; 3 Department of Community Medicine, All India Institute of Medical Sciences, Nagpur, Maharashtra, India;

**Keywords:** Diabetes mellitus (DM), diabetic kidney disease (DKD), urinary albumin-to-creatinine ratio (UACR), chronic kidney disease (CKD), diagnostic accuracy, rural population

## Abstract

Chronic kidney disease (CKD) is a major microvascular complication of diabetes in rural India, where access to diagnostic facilities is limited. 24-hour urine protein estimation, through the reference method is impractical for community screening. Therefore, it is of interest to evaluate the diagnostic accuracy of urinary albumin-to-creatinine ratio (UACR) for CKD screening among 201 known diabetic individuals from rural Nagpur. Elevated UACR was observed in 99% of participants, whereas 59% had abnormal 24-hour urine protein excretion. UACR demonstrated 100% sensitivity but low specificity (4%), with an overall diagnostic accuracy of 60% and a negative predictive value of 100%. UACR correlated significantly with glycaemic indices, eGFR and proteinuria and its screening utility improves when combined with serum creatinine and eGFR in resource-limited community settings.

## Background:

Diabetes mellitus (DM) is a worldwide epidemic; India contributes to one fifth of the global diabetes burden with an estimated prevalence of 9.1% among the adults [1]. Micro and macro vascular complications of DM usually go undetected especially in rural population, due to the lack of awareness & diagnostic facilities; diabetic kidney disease (DKD) is the more common of them [2]. According to guidelines provided by Indian Council for Medical Research (ICMR), regular monitoring for renal function is mandatory, but even at tertiary care centres less than 50% of the people adheres to it [3,4]. Hemodynamic changes occur as a result of the activation of the renin angiotensin-aldosterone and endothelin systems, resulting in increased systemic and intraglomerular pressure, causing hyper filtration and albuminuria [5]. Approximately 80% of End Stage Renal Disease (ESRD) is associated with DM, DKD also accounts for significant financial burden on health care systems [6]. DKD is diagnosed by detecting albuminuria and decreased Glomerular Filtration Rate (GFR) with evidence showing that DKD is reversible, if detected early [7]. Although the 24 hours urine sample is ideal to detect albuminuria, it is not a good screening test especially for rural and sub-urban region [8]. There has been a growing body of evidence that suggests a shift in the natural history of chronic kidney disease in patients with DM [9]. Normoalbuminuric chronic kidney disease in Diabetes is characterised by a reduction in glomerular filtration rate in the absence of albuminuria [10]. Non-albuminuric chronic kidney disease was also reported as the dominant presentation in non-communicable disease population in central India [10]. The American Diabetic Association (ADA) recommends use of Urinary Albumin-to-Creatinine Ratio (UACR) in a random spot urine collection and estimated GFR to screen for chronic kidney disease in Diabetic patients [11,12]. Therefore, it is of interest to report the burden and patterns (albuminuric and non-albuminuric) of chronic kidney disease in patients with Diabetes and yield of screening using UACR and eGFR at community level.

## Materials and Methods:

## Participants:

This community based cross-sectional analytical study was carried out among known subjects with DM on treatment currently were recruited from selected rural & sub-urban population of Khapri village at Nagpur, Maharashtra. The study was conducted after obtaining permission from Institute Human Ethical Committee (IHEC) of AIIMS Nagpur. The subjects were recruited after obtaining the informed consent in their own language: Assuming the sensitivity of UACR as 80% against 24 hours urine protein estimation as a reference standard with 10% absolute precision and the prevalence of albuminuria among patients with diabetes as 20%, alpha error 5% and design affect 2 the sample size estimated was 620. Considering the non-response rate for participation and 24 hours urine protein as 10% the sample size required would be 700. Khapri PHC caters to the population of around 30000. Assuming 7-9% prevalence of DM among adults (30 years and above) there are 1200 estimated diabetics in the targeted study area. From the recent Community Based Assessment Check List (CBAC) line list, DM patients on treatment were extracted. Patients who are suffering from hypertension, haematuria, fever and urinary tract infection were excluded on basis of relevant history, examination and investigation. Pregnant women were also excluded from this study. Subjects of DM with any other known secondary / specific causes, hereditary metabolic disorders or organ dysfunctions were excluded from this study. Known secondary/ specific causes of chronic kidney disease other than diabetes mellitus were excluded.

## Sample and data collection:

Patients with diagnosed Diabetes Mellitus (both type 1 and type 2) were identified through female health worker from the sub centre village. With the line list, the patients were approached in their household using structured proforma. 5 mL of venous blood sample was collected for estimation of fasting plasma glucose (FPG) and HbA1C. Two urine samples were collected from each subject with DM (24 hours sample and other random sample). Currently the diabetic kidney diseases are screened by estimation of 24 hours urine proteins, which is accepted as the preferred screening method. Sterile urine cans of 5L capacity, containing 5ml of 10% thymol in iso-propanol as preservative, were provided to each of the participant with the instructions card printed in their own language. It was also explained to them properly. In addition, Random / spot samples was collected in sterile urine container (10 ml/capacity) at the time of collection of blood sample for estimating urine albumin / creatinine ratio [13]. Urinary albumin was measured by Pyrogallol Red dye binding method while urinary creatinine by Jaffe's spectrophotometric method. For protein the Biuret test was used and the hexokinase method was used for glucose estimation. HbA1c was measured using reversed ion exchange chromatography. The samples were collected by a trained phlebotomist whose competency was assessed prior to the recruitment. The blood and urine samples were collected after obtaining prior informed consent (in patients own language). Sample collection was under the supervision of a medical officer. The samples collected were transported by our phlebotomist using sample transportation bag with temperature monitoring. The analysis was done at clinical chemistry laboratory, AIIMS Nagpur after getting the prior approval from the professor and head of the department.

## Study parameters:

Age, gender, duration of diabetes, history of smoking/smokeless tobacco, history of alcohol intake, history & duration of hypertension, FPG, HbA1c, serum creatinine, 24 hours urine proteins, UACR (with random & 24 hours urine samples), eGFR using CKD-EPI formula (Creatinine). All the biochemical analyses were performed by IFCC (International Federation of Clinical Chemistry and Laboratory Medicine) approved methods.

## CKD-EPI (Chronic Kidney Disease Epidemiology Collaboration) formula:

eGFR =141 x min (SCr/κ, 1)α x max (SCr /κ, 1)-1.209 x 0.993 Age x 1.018 [if female] x 1.159 [if Black] eGFR (estimated glomerular filtration rate) = mL/min/1.73 m_2_ SCr (standardized serum creatinine) = mg/dL κ = 0.7 (females) or 0.9 (males) α = -0.329 (females) or -0.411 (males) Min = indicates the minimum of SCr/κ or 1 Max = indicates the maximum of SCr/κ or 1 Age = years [14]. Link for online calculation: https://www.kidney.org/professionals/kdoqi/gfr-calculator. The Kidney Disease Improving Global Outcomes (KDIGO) 2020 Clinical Practice Guideline for Diabetes Management in Chronic Kidney Disease (CKD) will be used for staging CKD patients. It uses eGFR and UACR to categorize into 5 groups and 3 albuminuria categories (A1, A2 and A3) after 2 assessments of GFR and albuminuria.

## Statistical analysis:

Data was entered in EpiData and analysed using SPSS (v 21.0) software. Patient related demographic and clinical characteristics were summarized as frequencies and percentages. Diagnostic accuracy measures of sensitivity, specificity, positive predictive value and negative predictive value against the status based on 24 hours urine protein will be described as percentages with 95% CI. Association of glycaemic control measures (Fasting Blood sugar, HbA1C) and nephropathy related biochemical parameters will be described as intra cluster correlation with 95%.

## Results and Discussion:

**Figure 1:**
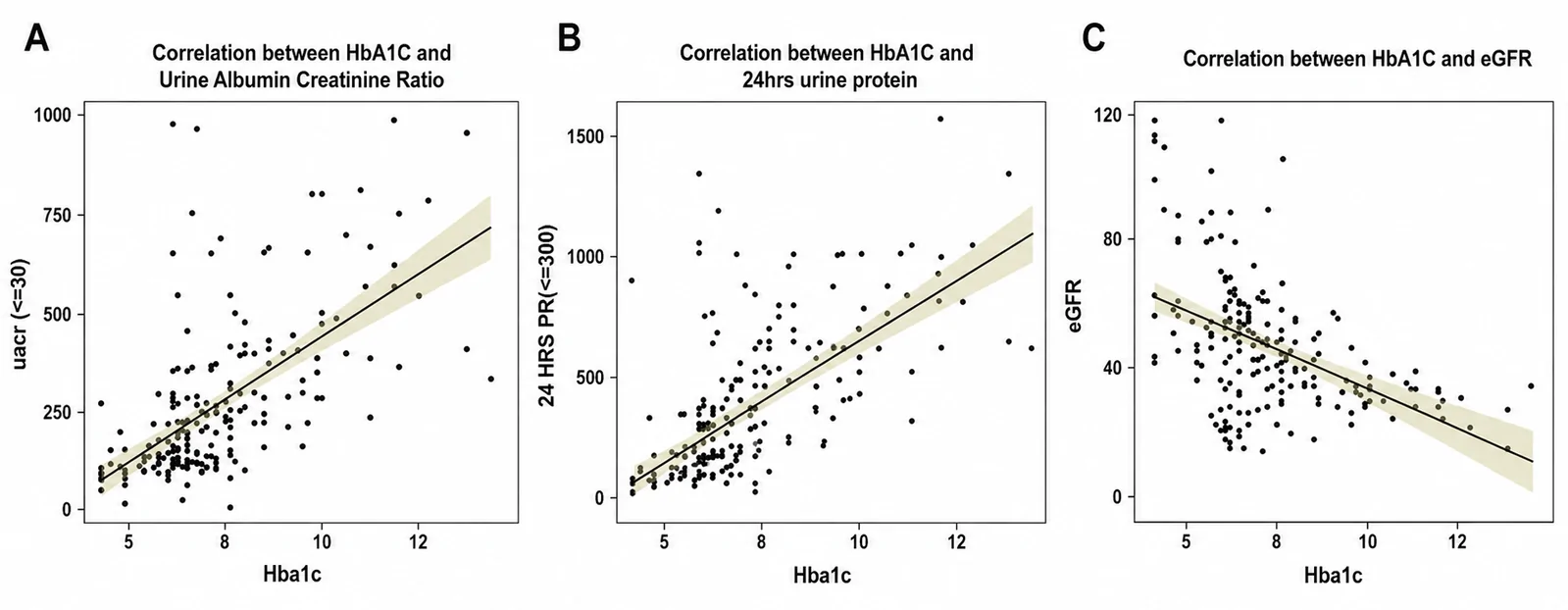
Scatter plots demonstrating associations between HbA1c and urinary albumin creatinine ratio (1A), 24 hours protein (1B) and eGFR (1C)

**Figure 2:**
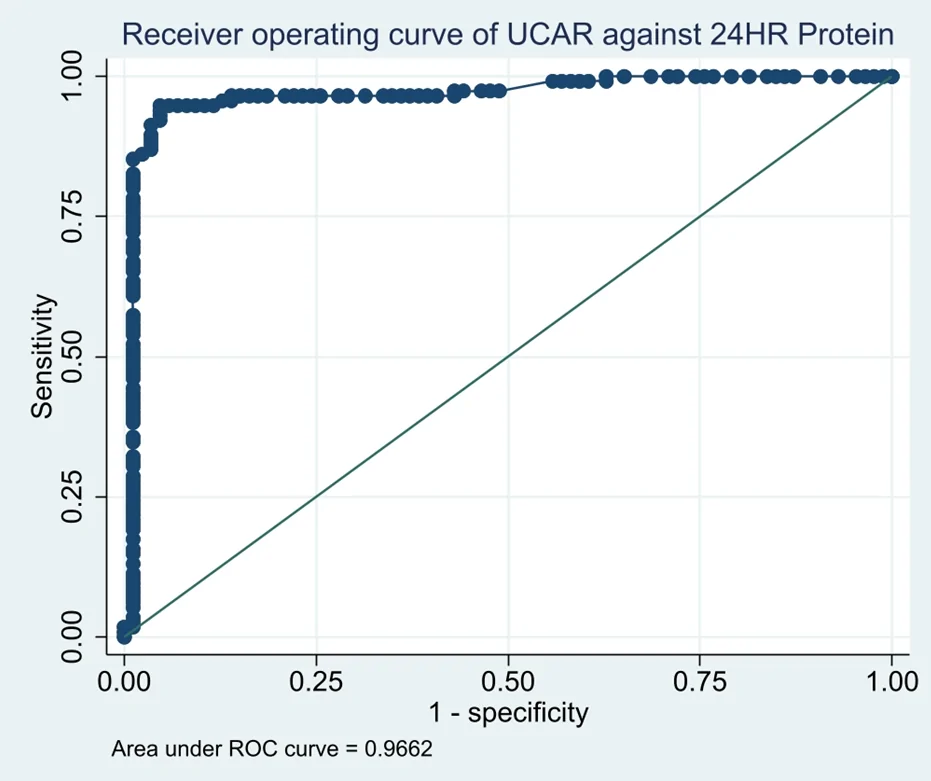
Receiver operating characteristic (ROC) curve of urinary albumin-to-creatinine ratio (UACR) for detection of nephropathy using 24-hour urinary protein excretion as the reference standard. UACR demonstrated excellent overall discriminative ability, with an area under the ROC curve (AUC) of 0.966, indicating high sensitivity for detecting abnormal 24-hour urinary protein excretion in diabetic subjects.

The demographic and clinical characteristics of the 201 study participants are summarized in Table 1 The cohort comprised 135 males (67%) and 66 females (33%), with a mean age of 50.89 ± 8.92 years. The distributions of all continuous variables were non-normal, as confirmed by the Shapiro-Wilk test. Mean HbA1c was 7.86 ± 1.59% and mean fasting plasma glucose was 170.29 ± 37.1 mg/dL, reflecting suboptimal glycaemic control. Mean serum creatinine was 2.46 ± 1.73 mg/dL, with a corresponding mean eGFR of 39.74 ± 22.81 mL/min/1.73 m^2^. A UACR value >30 mg/g creatinine and 24-hour urinary protein >300 mg/day were considered markers of nephropathy [2]. Elevated UACR was observed in 198 participants (99%), whereas abnormal 24-hour protein excretion was present in 118 participants (59%). Diagnostic accuracy analysis evaluated UACR as the index test against 24-hour urinary protein as the reference standard. Among the 201 participants, 118 were classified as diseased and 83 as non-diseased. UACR demonstrated 100% sensitivity but very low specificity (4%). Overall diagnostic accuracy was 60%, with a disease prevalence of 59%. The positive predictive value was 60%, while the negative predictive value was 100%. The positive likelihood ratio (LR+) was 1.04, indicating minimal rule-in value, whereas the negative likelihood ratio (LR-) was 0.00, indicating excellent rule-out capability. The Youden Index was 0.04, reflecting poor discriminatory performance. Although the diagnostic odds ratio was mathematically infinite due to absence of false negatives, its clinical relevance was limited by extremely low specificity (Table 2). Correlation analyses using Pearson's r and Spearman's rho demonstrated strong positive associations between HbA1c and UACR (r = 0.62) and a moderate positive correlation with 24-hour urinary protein (r = 0.65). A significant inverse correlation was observed between HbA1c and eGFR (r = -0.48), indicating declining renal function with worsening glycaemic control. Scatter plots further illustrated these linear relationships (Figure 1). ROC curve was plotted to assess the diagnostic significance of Fet-A and Cystatin-B. AUC was found to be 96.62% for UACR as shown in Figure 2.

This community-based cross-sectional study evaluated the utility of urinary albumin-to-creatinine ratio (UACR) as a screening tool for chronic kidney disease in known diabetic individuals from a rural Indian setting, using 24-hour urinary protein as the reference standard. The study population demonstrated poor glycaemic control, with a mean HbA1c of 7.86%, consistent with the known burden of inadequately controlled diabetes in rural communities. Poor glycaemic control is a key driver of diabetic kidney disease (DKD). Chronic hyperglycaemia promotes glomerular hyperfiltration, mesangial expansion and endothelial dysfunction, leading to albuminuria and progressive decline in eGFR [15]. In the present study, HbA1c showed strong positive correlations with both UACR and 24- hour proteinuria and an inverse correlation with eGFR, supporting established pathophysiological mechanisms. These findings reinforce the importance of monitoring renal biomarkers alongside glycaemic indices in diabetic populations. The 24-hour urine protein estimation is considered the reference standard for quantifying proteinuria but is cumbersome, time-consuming and prone to collection errors, particularly in community and rural settings. Consequently, spot urine tests such as UACR and protein-to-creatinine ratio (PCR) are widely used as practical alternatives [16,17]. Several studies have demonstrated good correlation between spot urine ratios and 24-hour protein excretion. Recent studies also demonstrate a significant correlation between spot urine protein-to-creatinine ratio and 24-hour urinary protein excretion, supporting its role as a practical alternative for proteinuria assessment [18]. A similar reasonable agreement was demonstrated between random UACR and 24-hour urine albumin excretion in diabetic patients [12]. In glomerular diseases such as IgA nephropathy, UACR has also shown strong concordance with 24-hour protein and superior prognostic value compared to PCR [19]. UACR offers the advantage of correcting for urine concentration by indexing albumin excretion to creatinine, which is relatively stable, thereby reducing the influence of hydration status [16]. International guidelines recommend UACR as the preferred initial test for albuminuria assessment in diabetes due to its sensitivity, simplicity and accessibility [15]. Despite this, the present study demonstrated markedly low specificity (4%) of UACR when compared with 24-hour urinary protein, resulting in a high false-positive rate. This finding contrasts with results from controlled hospital-based studies and highlights the challenges of real-world community screening. Spot urine albumin measurements are influenced by hydration, posture, physical activity, diurnal variation, diet and inter-day biological variability. Rasaratnam et al. reported significant within-individual variability in albuminuria among patients with type 2 diabetes, emphasizing that a single UACR measurement may not reliably represent persistent albuminuria [20]. Such variability is likely amplified in rural field settings where standardized early-morning sampling is difficult. Recent evidence also suggests that spot PCR may complement UACR. Gnanapraba et al. reported that although both ACR and PCR are useful screening tools, neither can fully replace 24-hour protein estimation due to modest agreement in some diabetic populations [21]. Similarly, Mahaseth *et al.* found limited agreement between spot PCR and 24-hour protein in patients with persistent glomerular proteinuria, recommending cautious interpretation alongside clinical context [22]. The diagnostic accuracy findings of the present study indicate that UACR has excellent sensitivity and perfect negative predictive value, supporting its role as an effective rule-out test for CKD in diabetic patients. A normal UACR reliably excludes significant proteinuria, making it suitable for large-scale community screening. However, the extremely low specificity and poor positive likelihood ratio underscore that elevated UACR values should not be used in isolation to confirm disease. Repeat testing or confirmation with 24-hour urine protein estimation remains essential, in line with guideline recommendations that persistent albuminuria must be documented on repeated measurements [20]. A key strength of this study is its community-based design in a rural Indian population, providing real-world evidence of UACR performance outside controlled clinical environments. The comprehensive assessment of glycaemic indices, eGFR and both spot and timed urine protein enhances its clinical relevance.

## Advancement to knowledge

This study provides important real-world evidence on the performance of urinary albumin-to-creatinine ratio (UACR) for screening diabetic kidney disease (DKD) in rural Indian populations, where data are scarce and diagnostic challenges are substantial. It highlights the significant presence of non-albuminuric chronic kidney disease; supporting the evolving understanding that CKD in diabetes may occur even in the absence of albuminuria and underscoring the importance of combined assessment using both UACR and estimated glomerular filtration rate (eGFR). The findings demonstrate that UACR possesses excellent sensitivity and negative predictive value, making it a reliable rule- out test for CKD in community-based settings. However, the markedly low specificity observed indicates a high false-positive rate, emphasizing that UACR should not be used as a standalone confirmatory test and must be interpreted with caution in field conditions. Additionally, the study reinforces the strong association between poor glycaemic control and renal dysfunction, as evidenced by significant correlations between HbA1c and nephropathy-related biomarkers. By providing community-based data, this study bridges the gap between g uideline recommendations and real-world practice, supporting a pragmatic two-step screening approach using UACR for initial screening followed by confirmatory testing. Overall, it advances current knowledge by redefining the role of UACR as an effective screening tool while highlighting its limitations in confirming disease in resource-limited rural settings.

## Conclusion:

UACR is a highly sensitive tool for ruling out CKD in diabetic patients but lacks specificity for confirming disease. Combining UACR with serum creatinine and eGFR enhances detection of both albuminuric and non-albuminuric CKD, making it suitable for community-level screening in resource-limited settings.

## Figures and Tables

**Table 1:** Clinical and demographic characteristics of study subjects:

**Variable**	**Mean ± S.D.**	**Median**	**Range**	**Normal (%)**	**Increased (%)**
Males (%)	135 (67%)				
Females	66 (33%)				
Age (years)	50.89 ± 8.92	49	32.00-78.00	-	-
HbA1c (%)	7.86 ± 1.59	7.4	5.40-13.50	-	-
Fasting Plasma Glucose (mg/dL)	170.29 ± 37.1	168.7	90.2-305.2	-	-
Creatinine (mg/dL)	2.46 ± 1.73	1.9	0.7-13.9	-	-
eGFR (mL/min/1.73 m^2^ BSA)	39.74 ± 22.81	24	4.0-116.0	-	-
UACR (mg/g of creatinine)	275.79 ± 201.3	223	9.0-988.0	3 (1%)	198 (99%)
Urinary 24 hours protein excretion (mg/day)	439.04 ± 269.39	365	122.0-1456.0	83 (41%)	118 (59%)

**Table 2:** Diagnostic accuracy of UACR versus 24 hours urinary protein excretion

**Decision Statistics**	**Estimate**	**95% CI Lower**	**95% CI Upper**
Sensitivity	100%	97%	100%
Specificity	4%	1%	10%
Positive predictive value	60%	52%	66%
Negative predictive value	100%	29%	100%
Positive likelihood ratio	1.04	1	1.08
Negative likelihood ratio	0	0	NaN
Diagnostic odds ratio	Inf	NaN	Inf
Youden's index	0.04	-0.02	0.1
Number needed to diagnose	27.67	-42.99	9.8
